# Dietary protein sources and disease severity, malnutrition and anthropometric measurements in cirrhotic patients 

**Published:** 2019

**Authors:** Fereshteh Pashayee-khamene, Hamed Kord-varkaneh, Mahdi Saber-firoozi, Behzad Hatami, Bahram Rashidkhani, Azita Hekmatdoost

**Affiliations:** 1 *Department of Clinical Nutrition and Dietetics, Faculty of Nutrition Sciences and Food Technology, National Nutrition and Food Technology Research Institute, Shahid Beheshti University of Medical Sciences, Tehran, Iran*; 2 *Digestive Disease Research Center, Digestive Disease Research Institute, Tehran University of Medical Science, Tehran, Iran*; 3 *Gastroenterology and Liver Diseases Research Center, Research Institute for Gastroenterology and Liver Diseases, Shahid Beheshti University of Medical Sciences, Tehran, Iran*

**Keywords:** Cirrhosis, Malnutrition, Dietary Protein

## Abstract

**Aim:**

To evaluate the association between dietary protein sources with disease severity, malnutrition and anthropometric measurements in cirrhotic patients.

**Background:**

Although the beneficial effects of protein and some amino-acids have been shown previously, no study has evaluated the effects of different dietary sources of proteins in patients with hepatic cirrhosis.

**Methods:**

In this cross-sectional study, dietary intakes of patients with hepatic cirrhosis were assessed using a valid and reliable food frequency questionnaire. The association between different dietary sources of proteins and nutritional status, anthropometric measurements, and disease severity were evaluated.

**Results:**

Muscle strength (MS) increased significantly in highest tertile of dairy and vegetable protein sources compared with the lowest one (p=0.045). Dietary dairy and vegetable protein intakes had a positive significant correlation with body weight, MS, visceral fat percentage (VFP), and triceps skin-fold thickness (TST), and negative significant correlation with malnutrition stage.

**Conclusion:**

Our results indicate that consumption of proteins from dairy and vegetable sources is associated with improvement in nutritional and anthropometric status of patients with hepatic cirrhosis. Further prospective studies are needed to confirm these results.

## Introduction

 Liver cirrhosis (LC) is the end stage liver disease with hepatocytes necrosis and hepatic fibrosis. LC is one of the public health issues with high rate of mortality (1, 2). Liver plays a key role in metabolism of carbohydrate, fat and protein. So, LC leads to notable protein energy malnutrition (PEM) due to dysfunction of hepatocytes in cirrhotic patients (1). PEM occurs in about 20% of well-compensated patients and in more than 60% of decompensate patients. PEM can determine the survival rate in liver cirrhosis(3). Thus, clinical management of liver cirrhosis includes diagnosis and intervention for PEM management (4). It has been shown that there is an association between PEM and prognosis of cirrhotic patients independently of liver function (5-7).

Subjective global assessment (SGA) is known as a suitable tool for nutritional assessment in patients with hepatic cirrhosis (8). Cirrhotic patients have imbalanced plasma amino-acid profile and protein intolerance when disease is decompensated (9). Decreased level of serum albumin and skeletal muscle volume are the indicators of visceral and muscular protein consecutively in liver cirrhosiss (4). Analysis of plasma amino acids in cirrhotic patients represents decreased branch chain amino acids(BCAA) and increased aromatic amino acids(AAA) that is important in pathogenesis of cachexia and encephalopathy (1, 4). Low level of plasma BCAA is due to increased removal and consumption of BCAA for ammonia detoxification by skeletal muscles and energy production. Decreased hepatic capacity to detoxify ammonia is the origin of hyperammonemiain cirrhotic patients. Thus, skeletal muscles and brain try to remove blood ammonia by using ammonia in the process of glutamine production from glutamate. BCAAs are essential for this synthesis (4). There are other factors that incorporate in reduction of plasma BCAA in these patients like activation of the branched-chain a-ketoacid dehydrogenase by cytokines and cortisol and by damaged reamination of branched-chain keto acids(10).

It has been shown that BCAA supplementation can improve impaired metabolism of albumin in cirrhotic patients(3). Thus, it is suggested that BCAAs supplementation improves hepatic status (4). Regulation of protein imbalance, liver regeneration, improved albumin level, improvement in mental and physical status and immune system are other positive effects of BCAA supplementation (4, 10, 11). On the other hand, the main dietary sources of BCAAs are dairy and vegetable proteins. Thus, we designed this study to evaluate the association between dietary protein sources with disease severity, malnutrition and anthropometric measurements in cirrhotic patients. 

## Methods

**Study design**

The study protocol was approved at National nutrition and Food Technology Research Institute (NNFTRI) ethics committee. Patients with at least 6 months of hepatic cirrhosis diagnosis were recruited from two Hepatology clinics in Tehran, Iran. After explanation of the study protocol, patients who agreed to participate in the study signed the inform consent form and enrolled in the study. The exclusion criteria were pregnancy for female patients, chronic renal or cardiac diseases, diabetes mellitus, pancreatic insufficiency, malignancies and acquired immuno deficiency syndrome. Finally, sixty eight patients were enrolled in this cross-sectional study from December 2016 to September 2018.

**Dietary assessment**

Dietary protein sources intakes were assessed using a validated Food Frequency questionnaire (FFQ) (12). Dietary protein sources were asked according to United States Department of Agriculture (USDA) serving sizes definition for each food item. Dairy items included milk, yogurt, cheese, yogurt drink, and kashk. Vegetable protein sources included legumes, and soya. Poultry, meat, and fish were considered as animal protein sources. 

**Nutritional status assessment**

Malnutrition was graded using SGA according to Destky et al report (8). The standard SGA includes nutritional evaluation of height, weight (current, before illness and weight variation in the previous 6 months), nutritional history (appetite, intake, gastrointestinal symptoms), physical examination assessment of fat loss, muscle wasting, and presence of ascites or encephalopathy, infections and renal insufficiency. Based on this evaluation, patients were classified prospectively into three groups: A: well-nourished B: moderately malnourished C: severely malnourished. 

**Disease severity assessment**

The severity of liver disease was assessed by the child-pugh and model for end-stage liver disease (MELD) classification. The score was calculated by serum albumin, total bilirubin, international normalized ratio (INR), and presence of ascites or encephalopathy. 

**Anthropometric measurements**

Anthropometric indices were measured accurately (13), Height was measured using a portable stadiometer to the nearest of 0.1 cm. Weight was measured without shoes and wearing light clothes using digital scales. Body mass index (BMI) was calculated by dividing weight in kg to squared height in m. Triceps skin fold thickness (TST) was measured with a Lange caliper. Mid arm circumference (MAC) was calculated from the right arm at mid-point equidistant from the acromion and olecranon, while the subject was in the upright position with arm flexed at 90°. The Mid arm muscle circumference (MAMC) was calculated as MAMC=MAC − (TST×0.3142) (2). 

Body composition including muscle mass percent (MMP), fat mass percent (FMP), and visceral fat percent (VFP) was measured using a Multi frequency bioelectrical impedance analysis (BIA) machine (Bioscan 920-2-S, UK). The participants were measured wearing light cloth with bare feet after being asked to empty their bladder. Muscle strength (MS) was measured using a Lite Hydraulic Hand Dynamometer. Right hand grip strength was measured three times, and the average of them was reported.

**Statistical analysis**

Data were analyzed using SPSS (Statistical Package For the Social Science) version.22. The quantitative variables were expressed as mean and standard deviation, and qualitative variables were shown by absolute and relative frequencies. The association between the severity of the disease, anthropometric measurements, and malnutrition with the consumption of different food groups was analyzed using chi-square or Fisher exact test. Logistic regression models were used to examine the association between different dietary protein sources and severity of malnutrition and illness. 

**Table 1:** General characteristics of the participants in the study based on tertiles of animal proteins and dairy and vegetable proteins

P Value*	dairy and vegetable proteins			P Value*	tertiles of animal proteins			
	tertile 3 (23)	tertile 2 (22)	Tertile1 (23)		tertile 3 (22)	tertile 2 (23)	Tertile1 (23)	
0.345	10.30±2.72	11.81±4.38	10.65±3.56	0.740	11.40±3.40	10.69±3.13	10.65±4.29	MELD
								CHILD-pugh
0.529	38.30%	31.90%	29.80%	0.695	31.90%	36.20%	31.90%	mild
	25%	35%	40%		35%	30%	35%	medium
	0%	0%	100%		0%	0%	100%	severe
								SGA
0.139	44.40%	37%	18.50%	0.420	33.30%	33.30%	33.30%	well-nourished
	30.30%	24.20%	45.50%		33.30%	39.40%	27.30%	moderately malnourished
	12.50%	50%	37.50%		25.0%	12.5%	62.5%	severely malnourished
0.74	55.65±11.92	58.04±10±26	50.34±11.85	0.898	55.40±12.78	53.78±11.68	54.73±11.01	Age(years)
0.027	18(36.7%)	19(38.8%)	12(24.5%)	0.120	18(36.7%)	18(36.7%)	13(26.5%)	Sex(male)
0.123	78.41±12.26	74.18±16.54	69.30±15.48	0.685	76.29±15.74	72.78±15.24	72.91±14.76	Weight(kg)
0.483	27.68±4.42	26.39±4.83	25.95±5.7	0.624	26.71±4.91	25.93±4.79	27.38±5.37	BMI (kg/m2)
0.580	1.08±0.54	0.92±0.49	0.94±0.60	0.426	1.04±0.65	0.86±0.34	1.05±0.59	TST
0.984	29.01±4.06	28.90±5.21	29.17±5.37	0.207	28.36±4.34	28.21±4.78	30.49±5.20	MAC
0.756	25.29±3.46	25.61±3.78	26.13±4.04	0.197	24.84±3.00	25.41±3.83	26.81±4.15	MAMC
0.045	68.91±23.48	59.65±22.78	51.42±21.75	0.266	63.92±22.74	63.52±21.78	53.80±25.30	MS
0.105	31.75±4.83	34.24±4.59	30.88±5.69	0.243	32.20±3.14	33.44±5.68	30.73±6.14	MMP
0.307	28.21±9.75	24.32±8.85	28.90±11.20	0.156	27.21±7.31	24.58±9.98	30.58±12.06	FMP
0.25	11.52±5.22	10.61±4.73	7.71±3.82	0.580	10.80±5.88	9.26±4.45	9.92±4.14	VFP

* Obtained from ANOVA for continuous variables and χ2 test for categorical variables; BMI, body mass index; TST: Triceps Skinfold Thickness; MAC: Mid arm circumference; MAMC: Mid arm muscle circumference; MS: Muscle strength; MMP: Muscle mass percent; FMP: Fat mass percent; VFP: Visceral fat percent

**Table 2:** Multiple linear regression analysis that shows the association between animal proteins and dairy and vegetable proteins and anthropometric variables

	animal proteins		dairy and vegetable proteins	
	β	P*	β	**P** *
MELD	0.176	0.196	-0.195	0.163
Weight(kg)	0.061	0.646	0.282	0.037
BMI(kg/m2)	0.042	0.759	0.244	0.076
TST	-0.064	0.633	0.327	0.017
MAC	-0.184	0.162	0.112	0.414
MAMC	-0.195	0.151	-0.018	0.902
MS	-0.068	0.493	0.353	0.000
MMP	-0.177	0.066	-0.176	0.080
FMP	0.157	0.158	0.187	0.104
VFP	0.081	0.526	0.326	0.012

* All variables were adjusted for age, sex, smoking, andalcohol. BMI, body mass index; TST: Triceps Skinfold Thickness ; MAC: Mid arm circumference; MAMC: Mid arm muscle circumference; MS: Muscle strength; MMP: Muscle mass percent; FMP: Fat mass percent; VFP: Visceral fat percent

**Table 3:** Odds ratios of disease severity and malnutrition based on the tertiles of protein consumption

Variables	tertiles of animal proteins			P trend	tertiles of animal proteins			P trend
	1	2	3		1	2	3	
Child-Pugh								
Crude model	1.00	0.66(0.18-2.34)	0.87(0.25-3.02)	0.824	1.00	0.72(0.21, 2.47)	0.43(0.11, 1.58)	0.205
*Model 1	1.00	0.52(0.13-2.01)	0.67(0.17-2.67)	0.335	1.00	0.53(0.13-2.04)	0.28(0.06-1.29)	0.102
* *Model 2	1.00	0.59 (0.14-2.52)	0.72(0.16-3.27)	0.714	1.00	0.57 (0.12-2.71)	0.22(0.04-1.20)	0.076
SGA								
Crude model	1.00	0.83(0.25-2.75)	0.77(0.23-2.57)	0.672	1.00	3.3(0.91-11.92)	1.1(0.34-3.55)	0.073
*Model 1	1.00	1.04(0.27-3.95)	1.21(0.30-4.89)	0.778	1.00	0.35(0.08-1.52)	0.34(0.07-1.54)	0.191
* *Model 2	1.00	1.20(0.28-5.08)	1.29(0.27-5.99)	0.747	1.00	0.13(0.01-0.99)	0.14(0.02-0.95)	0.074
MELD								
Crude model	1.00	1.20(0.36-3.99)	2.70(.80-9.06)	0.106	1.00	1.30(0.40-4.20)	0.83(0.25-2.70)	0.767
*Model 1	1.00	1.16(0.30-4.40)	3.55(0.86-14.57)	0.74	1.00	1.64(0.43-6.28)	1.32(0.31-5.59)	0.726
* *Model 2	1.00	1.43(0.34-6.07)	4.27(0.87-20.83)	0.067	1.00	1.002(0.21-4.63)	0.84(0.18-3.89)	0.809

* Model 1: adjusted for BMI and energy intake;

** Model 2: adjusted for age, sex, alcohol, smoking, body mass index, and energy intake

**Table 4:** Dietary protein sources intakes in categories of CHILD-pugh and SGA. Data are analyzed using one-way ANOVA. SGA: subjective global assessment

	CHILD-pugh			SGA			
	Mild (n=47)	Moderateand Severe (n=21)	P-Value	Well-nourished (n=27)	Moderately malnourished (n=33)	Severely malnourished (n=8)	P-Value
Animal proteins intake *	4.09±2.68	4.49±3.5	0.69	4.13±2.52	4.52±3.32	3.26±2.56	0.54
Dairy and vegetable proteins intakes *	4.26±2.39	3.38±1.73	0.23	4.50±2.24	3.67±2.31	3.40±1.68	0.27

* serving±SE

## Results

The mean±SD of participants’ weight (kg), BMI (kg/m^2^), age (year), total intake of protein (gram/day), and energy (kcal/day) and MELD score were 74±15, 26.6±5, 54±11, 110±46, 2689±1223, and 10.9±3.6, respectively. Anthropometric, demographic, and laboratory data across tertiles of dietary protein sources of patients are shown in Table 1. As it is shown, muscle strength (MS) increased significantly in highest tertile of dairy and vegetable protein sources compared with the lowest one (p=0.045). Moreover, men more likely consumed dairy and vegetable protein sources in comparison to women (p=0.027). There was no other significant trend in tertiles of either animal protein sources or dairy and vegetable protein sources (p>0.05). All patients with severe cirrhosis were in the first tertile of protein intakes. 

Table-2 indicates the correlation of anthropometric, disease stage, and malnutrition indices with dietary protein sources intake. As it is shown, dietary dairy and vegetable protein intakes had a positive significant correlation with body weight (p=0.03), MS (p<0.001), VFP (p=0.01), and TST (p=0.01).

The prevalence of moderate and severe malnutrition in the study participants was 48%, and 12% respectively. Thirty one percent of participants were classified in child scores of B or C. 

Table-3 shows the odds ratios (OR) of disease severity and malnutrition based on the tertiles of protein consumption. Dietary protein sources did not alter significantly risk of disease severity and malnutrition. Adjusting for BMI, and energy intake in first model did not affect the trend. Even after adjusting for age, sex, alcohol, smoking, body mass index, and energy intake in model 3, no significant trend was observed in severity of disease and malnutrition across tertiles of dietary protein intake sources.

Different dietary protein sources were consumed more in those who were in lower stages of SGA and Child pugh; however, the trend was not significantly different (Table-4). As it is shown in table 4, patients in mild stages of CHILD-pugh classification had less intake of proteins from animal sources (4.09 serving/day), rather than dairy and vegetable sources (4.26 serving/day), while patients with moderate to severe disease had more intakes of proteins from animal sources (4.5 serving/day), rather than dairy and vegetable sources (3.38 serving/day). While both well-nourished and severely malnourished patients got more proteins from dairy and vegetable sources, the total amount of dietary protein was significantly lower in severely malnourished patients (p=0.049).

## Discussion

To our knowledge, this is the first report on the association between different dietary sources of protein intakes and disease severity, malnutrition, and anthropometric measurements in cirrhotic patients. Our results have shown that higher intakes of protein from dairy and vegetable sources are correlated with better status of anthropometric variables and malnutrition.

Only few studies have shown that nutritional interventions can be effective in the treatment of hepatic encephalopathy (14); however, high prevalence of malnutrition has been shown previously. Inadequate oral intake, metabolic disturbances, malabsorption, and decreased capacity of the liver to store nutrients and dietary restrictions imposed by the family are known as the aetiologies of malnutrition in these patients. Protein depletion is the main component of malnutrition in cirrhotic patients, which increases with greater disease severity and is associated with loss of skeletal muscle function (15). There is some evidence that BCAAs consumption improves nutritional status, anthropometric variables, and disease stage in patients with cirrhosis (4, 7, 16, 17). The main dietary sources of BCAAs are dairy and vegetable proteins; however, there is limited studies evaluating the effects of different dietary protein sources on cirrhosis and its related complications (18). 

A cross-over study has shown that feeding a diet rich in vegetable proteins significantly ameliorates mental state in patients with chronic hepatic encephalopathy under optimum lactulose treatment, and improves nitrogen balance (19). Moreover, a recent study has reported that ssupplementation with BCAAs plus a high-fiber, high-protein diet is a safe intervention in patients with cirrhosis. It increased muscle mass without rise in the levels of ammonia or glucose, and hepatic encephalopathy (20). Although these studies did not compare the main dietary sources of BCAAs with other protein sources, their results are consistent with our findings. 

Our results indicated that dairy and vegetable protein sources consumption improve nutritional status and muscle strength in cirrhotic patients in a cross-sectional analysis. If these associations confirm in prospective studies, they can be used in preparing nutritional guidelines for cirrhotic patients to improve their survival and reduce the comorbidities (21, 22).

This study has some strengths; using BIA for assessment of body composition is one of advantages of this study. BIA is a noninvasive and accurate method of estimating body composition (23); moreover, using dynamometer for measurement of grip strength as a sensitive and important index for evaluating nutritional status of patients was another advantage of this study (24). Finally, the main advantage of this study is that it evaluated dietary sources of proteins, which can be consumed in foods, which are available and affordable for most of the patients.

There are some limitations in this study; the study design was cross-sectional, which cannot show cause-effect relationship. Thus, we recommend further prospective studies to confirm these results. Presence of ascites and edema is a limitation for measuring body composition using BIA. Using FFQ for dietary assessment is prone to recall bias; however, we used a valid and reliable FFQ, and expert interviewers to minimize this limitation. Although objective measures were used for assessment of malnutrition some degree of observation bias might be involved while measuring anthropometric measurements.

In conclusion, our results indicate that consumption of proteins from dairy and vegetable sources is associated with improvement in nutritional and anthropometric status of patients with hepatic cirrhosis. Further prospective studies are needed to confirm these results.
